# Supplementary material for: Tricyano-Methylene-Pyridine Based High-Performance Aggregation-Induced Emission Photosensitizer for Imaging and Photodynamic Therapy
Source: Molecules. 2022 Nov 17;27(22):7981. doi: 10.3390/molecules27227981 (PMC9697965; doi:10.3390/molecules27227981)
Supplement: Supplementary file 1 [file molecules-27-07981-s001.zip › molecules-2028196-supplementary.pdf]

Tricyano-methylene-pyridine based high-performance aggregation-induced emission photosensitizer for imaging and photodynamic therapy

Xupeng Wu†, Zhirong Zhu†, Zhenxing Liu, Xiangyu Li, Tijian Zhou, Xiaolei Zhao, Yuwei Wang, Yiqi Shi, Qianqian Yu, Wei-Hong Zhu, Qi Wang*

Shanghai Key Laboratory of Functional Materials Chemistry, Key Laboratory for Advanced Materials and Institute of Fine Chemicals, Joint International Research Laboratory of Precision Chemistry and Molecular Engineering, Feringa Nobel Prize Scientist Joint Research Center, Frontiers Science Center for Materiobiology and Dynamic Chemistry, School of Chemistry and Molecular Engineering, East China University of Science and Technology, Shanghai 200237, China.

E-mail: wangqi@ecust.edu.cn.

Keywords: aggregation-induced emission, photosensitizer, ROS generation, bioimaging

Catalog

1 Experimental section 2
2 Quantum yield of TCM-Et and TCM-Ph 6
3 Particle size and morphology of TCM-Et and TCM-Ph 6
4 Total ROS generation evaluation of TCM-Ph 7
5 $^1O_2$ generation evaluation of TCM-Ph 8
6 Other types of ROS generation evaluation of TCM-Ph 8
7 PL spectra of compounds in solutions at 77 K 9
8 Particle size and morphology of TCM-Ph NPs 9
9 Total ROS generation evaluation of TCM-Ph NPs 10
10 Confocal images of HeLa cells incubated with TCM-Ph 10
11 Computational details 11

1 Experimental section

1.1 Materials and instruments

Hydrochloric acid (HCl), ethylamine aqueous solution and anhydrous potassium carbonate (K_2CO_3) were obtained from Sigma-Aldrich (Shanghai, China). Piperidine and sodium bicarbonate ($NaHCO_3$) were obtained from Macklin (Shanghai, China). Tetrahydrofuran (THF), ethyl acetate (EA), petroleum ether (PE), methanol (MeOH) dichloromethane (DCM), dimethyl sulfoxide (DMSO) and acetonitrile (MeCN) were obtained from General-Reagent (Shanghai, China). All solvents and raw materials were purchased from commercial suppliers in analytical grade and used without purification unless special noted.^{1}H and ^{13}C NMR spectra in deuterium generation reagent were obtained with a Bruker AvanceIII 400 MHz NMR spectrometer using TMS as an internal standard. High resolution mass spectrometry (HRMS) spectra were measured with a Waters LCT Premier XE spectrometer. UV-Vis absorption and fluorescence spectra were recorded on an Agilent Cary 60 spectrophotometer and Varian Cary Eclipse fluorescence spectrophotometer, respectively (10 × 10 mm quartz cuvette). Dynamic light scatting (DLS) and Zeta potential experiments were conducted with Zetasizer Nano-ZS (Malvern Instruments, Worcestershire, UK). TEM images were captured on a JEOL JEM-1400 transmission electron microscope. Confocal fluorescence images were performed on confocal laser scanning microscope (CLSM, Leica confocal microscope TCS SPS CFSMP).

1.2 Synthesis route

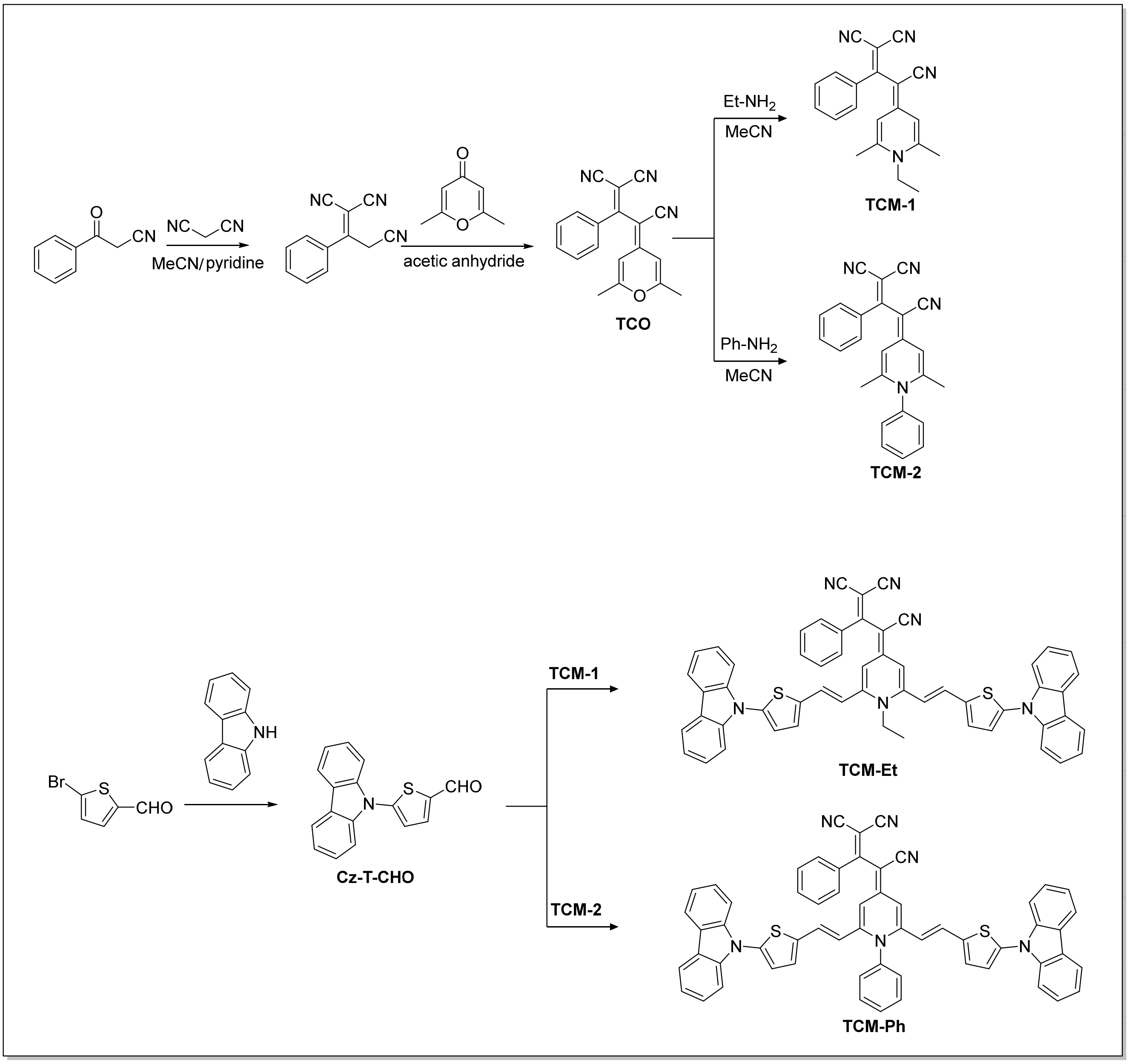

Scheme S1. Synthetic route of compounds TCM-Et and TCM-Ph

The synthesis of TCM is according to our previous reported work [36].

1.2.1 Synthesis of TCM-1

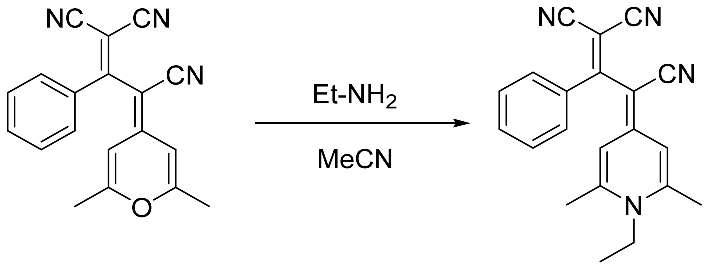

TCO (1 g, 3.34 mmol) was dissolved in acetonitrile (15 mL), then 4 mL aniline was added. The mixture was then stirred at 75°C for 12 h under argon protection. The solvent was removed under reduced pressure, and the crude product was separated by silica gel column chromatography (DCM: MeOH = 400:1) to afford the desired product TCM-1 (355 mg, 0.4 mmol), yield: 32.6%. ^{1}H NMR (400 MHz, DMSO-d_6, ppm): δ 7.60-7.52 (m, 1H, Ph-H), 7.52-7.45 (m, 4H, Ph-H), 6.96 (s, 2H, alkene-H), 4.25 (q, 2H, J = 7.2 Hz, N-CH_2-CH_3), 2.58 (s, 6H, -CH_3), 1.31 (t, 3H, J = 7.2 Hz, N-CH_2CH_3). ^{13}C NMR (100 MHz, DMSO-d_6, ppm): δ 165.44, 151.62, 150.90, 136.78, 131.04, 130.10, 128.79, 120.80, 119.97, 118.59, 117.13, 79.23, 58.15, 45.59, 20.01, 13.42. Mass spectrometry (ESI positive ion mode for $[M+Na]^+$): Calcd. for $C_{21}H_{18}N_4Na$: 349.1429; found: 349.1428.

1.2.2 Synthesis of Cz-T-CHO

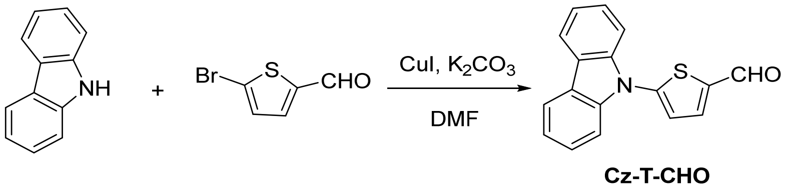

Cz (5 g, 29.90 mmol), 5-Bromothiophene-2-carbaldehyde (6.85 g, 35.86 mmol), CuI (0.57 g, 2.92 mmol) and dry K_2CO_3 (6.19 g, 44.79 mmol) were dissolved in dry DMF (25 mL). The mixture was then stirred at 140°C for 24 h under argon protection. After cooling, the reaction solution was poured into NaCl aqueous solution (100 mL), and then extracted with DCM three times (100 mL), at last crude product was separated by silica gel column chromatography (PE: DCM = 5:1) to afford the desired product Cz-T-CHO (3.52 g, 12.7 mmol),

yield: 42.4%.^{1}H NMR (400 MHz, $CDCl_3$, ppm): δ 9.95 (s, 1H, -CHO), 8.11 (d, 2H, J = 7.7 Hz, Ar-H), 7.88 (d, 1H, J = 4.0 Hz, thiophene-H), 7.67 (d, 2H, J = 8.2 Hz, Ar-H), 7.51-7.44 (m, 2H, Ar-H), 7.39-7.31 (m, 3H, Ar-H). ^{13}C NMR (100 MHz, $CDCl_3$, ppm): δ 182.84, 148.63, 140.55, 139.71, 136.10, 126.71, 124.25, 123.67, 121.71, 120.46, 110.34. Mass spectrometry (ESI positive ion mode for $[M+H]^+$) : Calcd. for $C_{17}H_{12}NOS$: 278.0640; found: 278.0616.

1.2.3 Synthesis of TCM-Et

TCM-1 (200 mg, 0.61 mmol) and Cz-THO (1020 mg, 3.68 mmol) were dissolved in acetonitrile (15 mL), then 0.6 mL piperidine was added. The mixture was then stirred at 95°C for 12 h under argon protection. The solvent was removed by filtration, and the crude product was separated by silica gel column chromatography (DCM: MeOH = 400:1) to afford the desired product TCM-Et (201 mg, 0.24 mmol), yield: 38.8%.^{1}H NMR (400 MHz, DMSO-d_6, ppm): δ 8.26 (d, 4H, J = 7.6 Hz, Ar-H), 7.75-7.70 (m, 1H, Ar-H), 7.68 (d, 2H, J = 3.6 Hz, thiophene-H), 7.66-7.55 (m, 8H, Ar-H), 7.55-7.48 (m, 4H for Ar-H, 2H for alkene-H), 7.40-7.33 (m, 4H, Ar-H), 7.20-7.17 (m, 2H, Ar-H), 7.16-7.13 (d, 2H, J = 15.6 Hz, alkene-H), 7.07 (s, 2H, Ar-H), 4.42 (q, 2H, J = 6.8 Hz, N-CH_2-CH3), 1.31 (t, 3H, J = 7.0 Hz, N-CH_2-CH_3). ^{13}C NMR (100 MHz, DMSO-d_6, ppm): δ 165.69, 151.46, 148.76, 140.54, 139.86, 137.42, 136.40, 132.79, 131.30, 130.45, 130.36, 129.37,126.79, 125.74, 123.13, 121.27, 120.64, 118.41, 118.16, 117.82, 116.69, 110.16, 79.95, 59.75, 46.05, 14.28. Mass spectrometry (ESI positive ion mode for $[M+H]^+$): Calcd. for $C_{55}H_{37}N_6S_2$:845.2521; found: 845.2517.

1.2.4 Synthesis of TCM-Ph

TCM-2 (150 mg, 0.40 mmol) and Cz-THO (889 mg, 3.21 mmol) were dissolved in acetonitrile (15 mL), then 0.5 mL piperidine was added. The mixture was then stirred at 95°C for 12 h under argon protection. The solvent was removed under reduced pressure, and the crude product was separated by silica gel column chromatography (DCM: PE = 4:1) to afford the desired product TCM-Ph (156 mg, 0.17 mmol), yield: 43.6%. ^{1}H NMR (400 MHz, DMSO-d_6, ppm): δ 8.21 (d, 4H, J = 7.6 Hz, Ar-H), 7.71-7.64 (m, 4H, Ar-H), 7.64-7.51 (m, 4H, Ar-H), 7.51-7.41 (m, 12H, Ar-H), 7.41-7.37 (m, 2H, Ar-H), 7.37-7.31 (m, 4H, Ar-H), 7.31-7.26 (m, 2H,

alkene-H), 7.23 (s, 2H, Ar-H), 5.95 (d, 2H, J = 15.6 Hz, alkene-H). ^{13}C NMR (100 MHz, DMSO-d_6, ppm): δ 166.18, 152.18, 148.03, 140.47, 139.66, 137.46, 137.01, 136.39, 131.41, 131.23, 130.84, 130.53, 130.37, 129.42, 127.79, 126.75, 126.18, 123.08, 121.25, 120.59, 119.06, 118.11, 118.02, 116.40, 115.18, 110.03, 80.45, 61.55. Mass spectrometry (ESI positive ion mode for $[M+H]^+$): Calcd. for $C_{59}H_{37}N_6S_2$: 893.2521; found: 893.2512.

1.3 Transmission electron microscopy imaging of TCM-Et, TCM-Ph and TCM-Ph NPs

10 μL TCM-Et, TCM-Ph (10 μM in 99% water) and TCM-Ph NPs (10 μM based on TCM-Ph) and was added onto a carbon-coated copper grid, followed by drying overnight at room temperature. The TEM imaging was performed on JEOL JEM1400 with an accelerating bias voltage of 100 kV.

1.4 Cell culture

Human epithelioid cervical carcinoma (HeLa) cells were purchased from the Institute of Cell Biology (Shanghai, China). Cells were propagated in cell culture flask at 37 °C under humidified 5% CO2 atmosphere. Dulbecco's modified eagle medium (DMEM, GIBCO/Invitrogen, Camarillo, CA, USA) was supplemented with 1% penicillin-streptomycin (10,000 U mL-1 penicillin, and 10 mg mL-1 streptomycin, Solarbio life science, Beijing, China) and 10% fetal bovine serum (FBS, Biological Industry, Kibbutz Beit Haemek, Israel).

2 Quantum yield of TCM-Et and TCM-Ph

Table S1. Absolute quantum yield of TCM-Et and TCM-Ph in solvent (THF/Water) and solid state

	Φ_{Water}[a]	Φ_{THF}[a]	Φ_{solid}[b]
**TCM-Et**	**2.23%**	**0.01%**	**2.5%**
**TCM-Ph**	**1.89%**	**0.01%**	**1.3%**

[a] Absolute quantum yield was measured in 99% (Vol%) water and THF, concentration of TCM-Et and TCM-Ph was 10 μM. Absolute quantum yield in water and THF was measured by SpectrumTEQ-PL (QEPro). [b] Absolute quantum yield was measured by HAMAMATSU Quantaurus-QY C11347-11.

3 Particle size and morphology of TCM-Et and TCM-Ph

A B

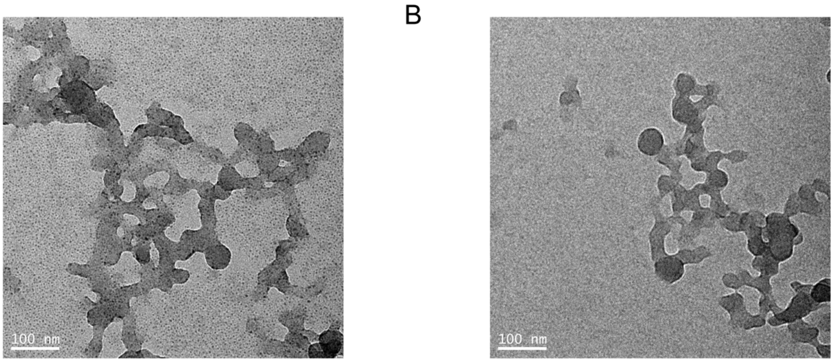

Figure S1. TEM images of (A) TCM-Et and (B) TCM-Ph.

4 Total ROS generation evaluation of TCM-Ph

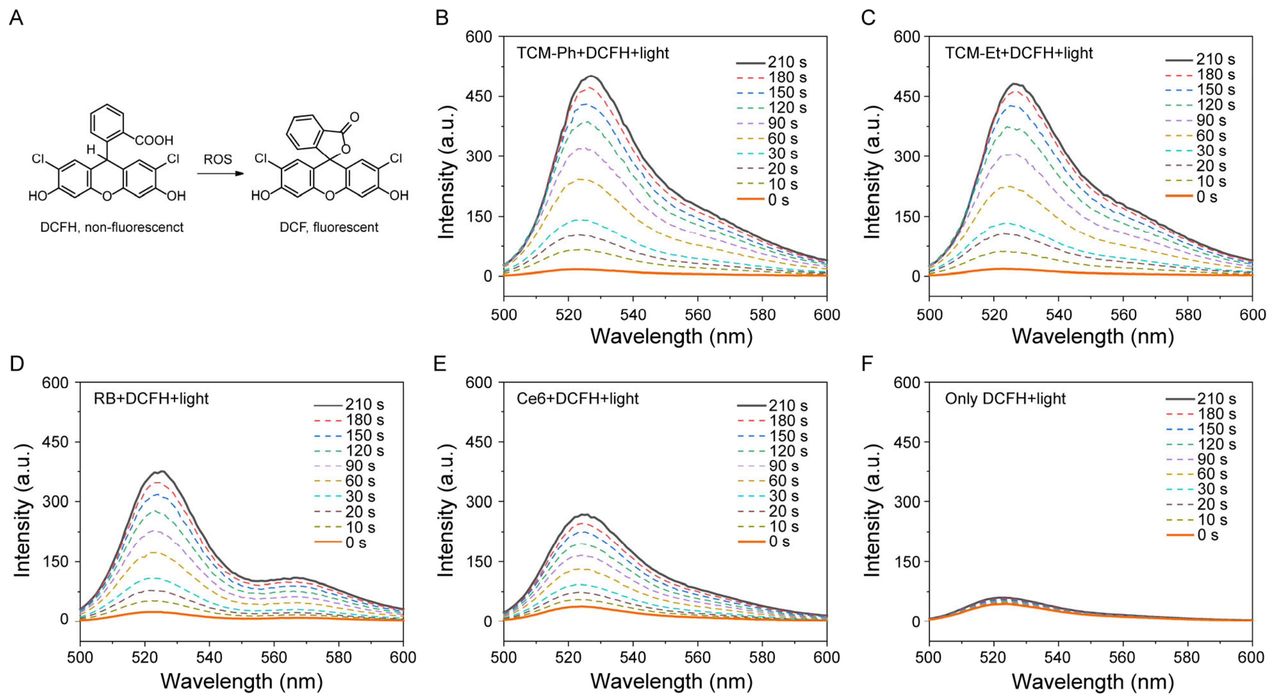

Figure S2. (A) Total ROS detecting mechanism by 2,7-dichlorodihydrofluorescein (DCFH). Total ROS generation by (B) TCM-Ph, (C) TCM-Et, (D) RB, (E) Ce6 (10 μM), (F) blank in water using DCFH (40 μM) as indicator, λ_{ex} = 488 nm.

5 $^1O_2$ generation evaluation of TCM-Ph

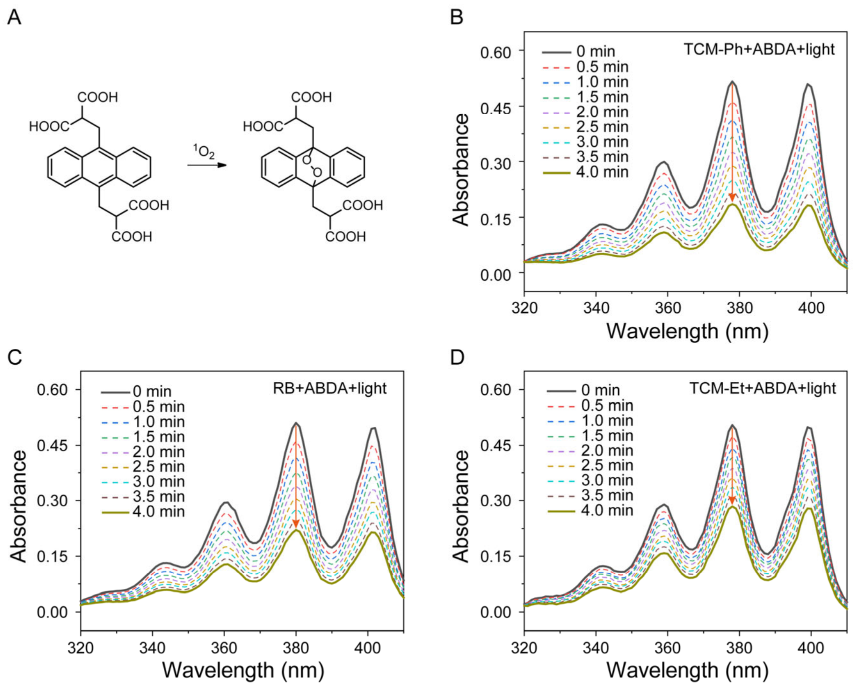

Figure S3. (A) $^1O_2$ detecting mechanism by indicator 9,10-anthracenediyl-bis(methylene) dimalonic acid (ABDA). Detection of $^1O_2$ produced by compounds (B) TCM-Ph, (C) RB, and (D) TCM-Et (10 μM) in water using ABDA (50 μM) as indicator.

6 Other types of ROS generation evaluation of TCM-Ph

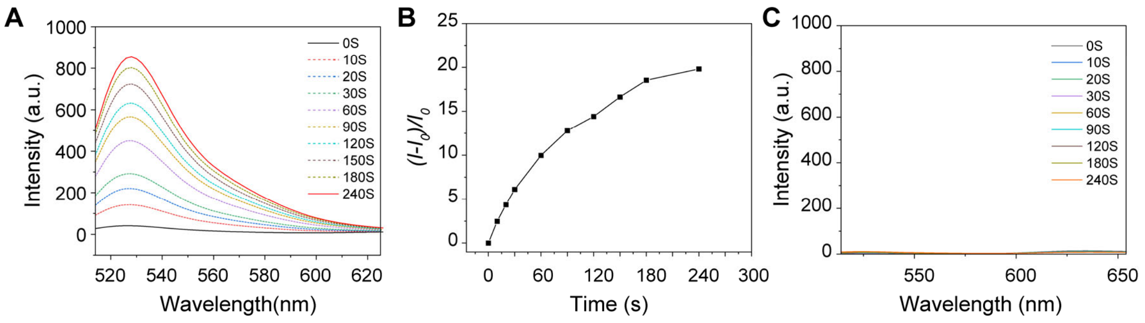

Figure S4. (A) Detection of $O_2^{\cdot-}$ produced by TCM-Ph, indicator: dihydrorhodamine 123 (DHR123). (B) The plot of relative PL intensity (I-I_0)/ I_0 at 530 nm versus the different irradiation time for figure A, where I_0 is the fluorescence value of the mixture at 530 nm before illumination, and I is the fluorescence value of the mixture at 530 nm after illumination, excitation wavelength is 488 nm. (C) Detection of $OH^{\cdot}$ produced by TCM-Ph, indicator: hydroxyphenyl fluorescein (HPF).

7 PL spectra of compounds in solutions at 77 K

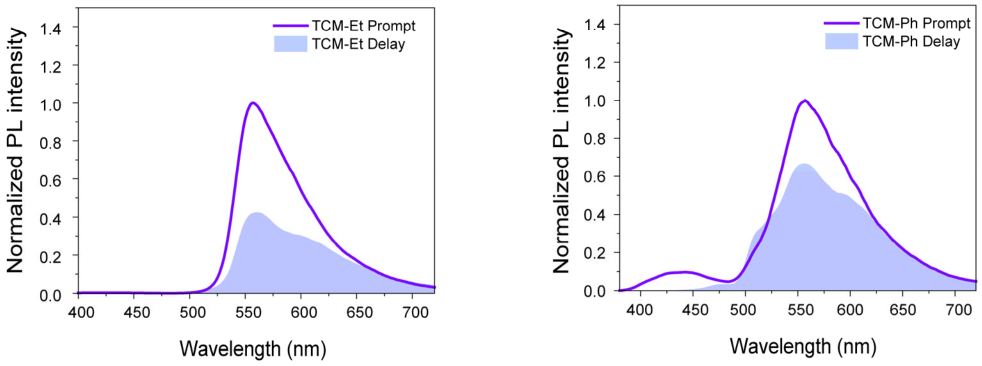

Figure S5. PL spectra of compounds in solutions at 77 K. The prompt (line) and delayed (color zone) PL spectra in 2-methyl-tetrahydrofuran solutions for TCM-Et and TCM-Ph, λ_{ex}: 365 nm. The results from E = h v = ħ c/λ, where λ was from the 0–0 peaks or the onsets of the emission bands, ħ was the reduced Planck's constant, c was the speed of light, and E was the energy level of S_1 or T_1.

8 Particle size and morphology of TCM-Ph NPs

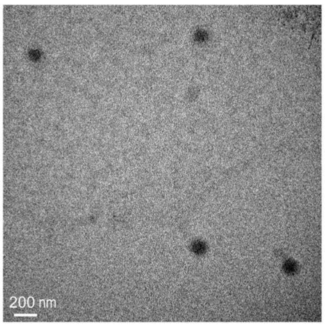

Figure S6. TEM image of TCM-Ph NPs.

9 Total ROS generation evaluation of TCM-Ph NPs

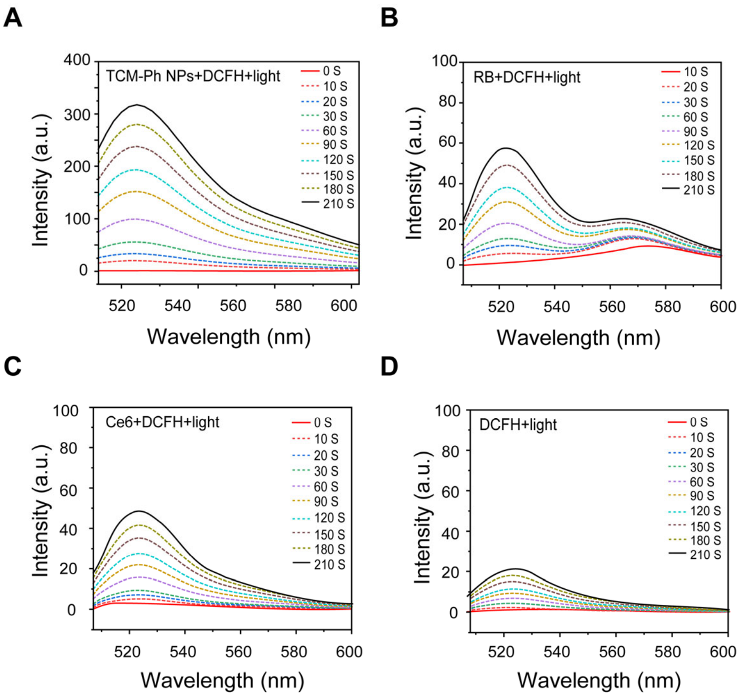

Figure S7. (A) Detection of total ROS produced by TCM-Ph NPs (10 μM based on TCM-Ph), (B) RB, (C) Ce6, (D) blank in water using DCFH (40 μM) as indicator, λ_{ex} = 488 nm.

10 Confocal images of HeLa cells incubated with TCM-Ph

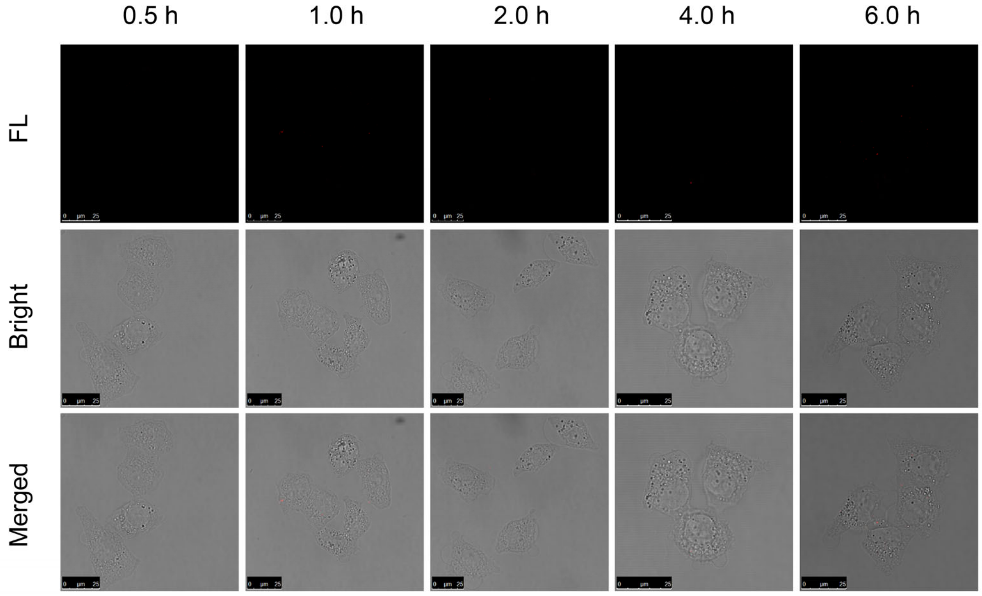

Figure S8. CLSM of HeLa cells stained with TCM-Ph (10 μM) for different times. Red channel from TCM-Ph, λ_{ex} = 514 nm, λ_{em} = 550-700 nm. Scale bar: 25 μm.

11 Computational details

All calculations were carried out with the Gaussian 16 software. The M06-2X functional was adopted for all calculations. For geometry optimization calculations, the def2-SV basis set was used, and the optimal geometry for each compound was determined. The excited states were calculated with linear response time-dependent DFT (TD-DFT) at the optimized ground state geometry with TZVP basis set. The SMD implicit solvation model was used to account for the solvation effect of DMSO. Grimme's DFT-D3 dispersion correction was used to improve calculation accuracy.

Table S2. Calculated energy of the singlet (S) and triplet (T) excited states

		1	2	3	4	5	6
TCM-Et	S	2.9179	3.1997	3.5891	3.7955	3.9942	4.0275
	T	2.2008	2.4137	2.4760	3.2827	3.4366	3.5138
TCM-Ph	S	2.8662	3.0486	3.4910	3.6871	3.8948	3.9822
	T	2.1827	2.2798	2.3737	3.2560	3.3571	3.4035

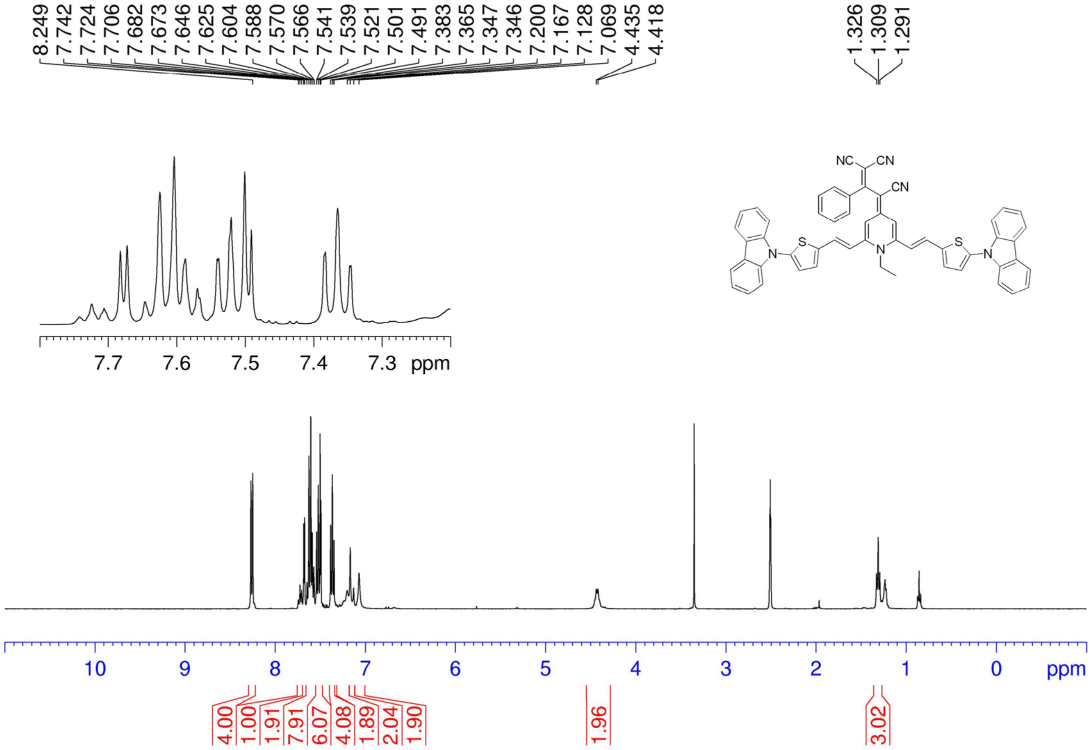

Figure S9. ^{1}H NMR spectrum of TCM-Et.

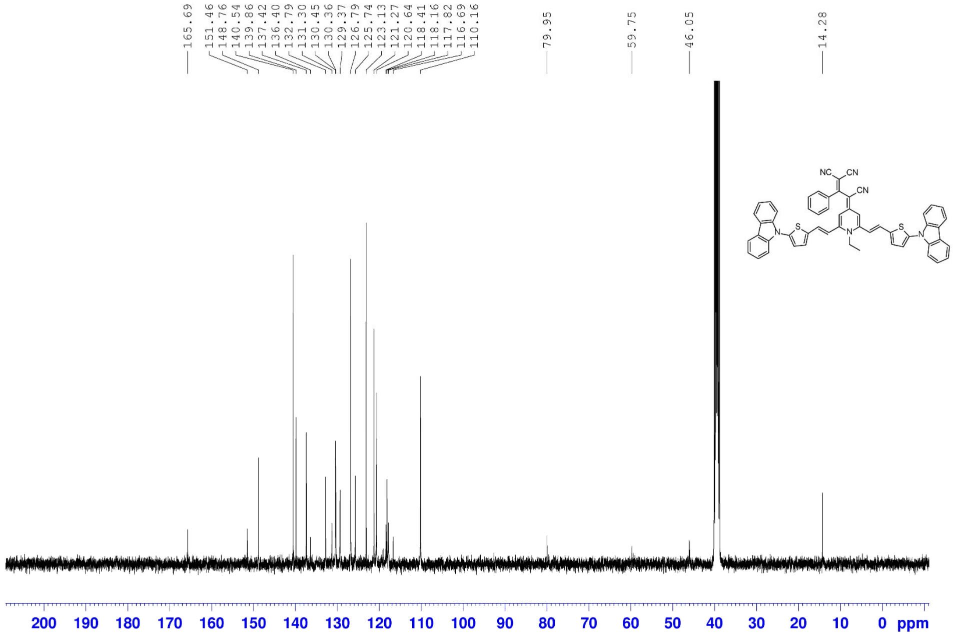

Figure S10. ^{13}C NMR spectrum of TCM-Et.

Single Mass Analysis
Tolerance = 5.0 PPM / DBE: min = -1.5, max = 50.0
Element prediction: Off
Number of isotope peaks used for i-FIT = 2

Monoisotopic Mass, Even Electron Ions
16 formula(e) evaluated with 1 results within limits (up to 50 closest results for each mass)
Elements Used:
C: 0-55 H: 0-74 N: 0-6 S: 0-2
WH-ZHU
ZW-LZX-214 100 (1.135) Cm (100:101) 1: TOF MS ES+ 1.10e+002

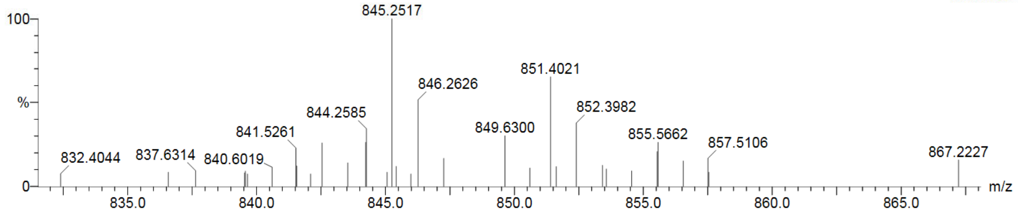

Mass	Calc. Mass	mDa	PPM	DBE	i-FIT	i-FIT (Norm)	Formula
Minimum:				-1.5			
Maximum:		5.0	5.0	50.0			
845.2517	845.2521	-0.4	-0.5	40.5	19.9	0.0	C55 H37 N6 S2

Figure S11. HRMS spectrum of of TCM-Et.

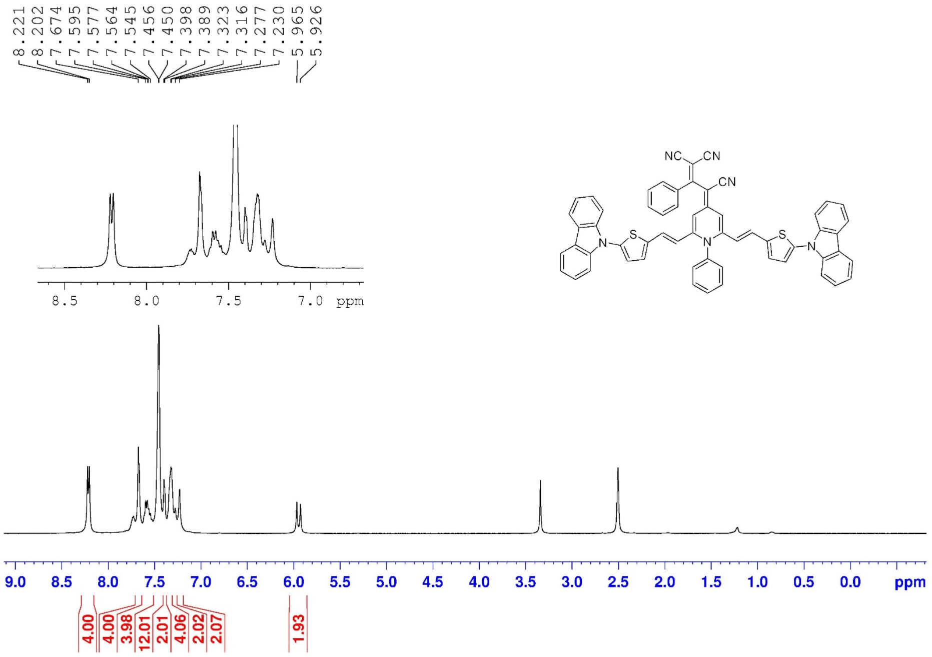

Figure S12. ^{1}H NMR spectrum of TCM-Ph.

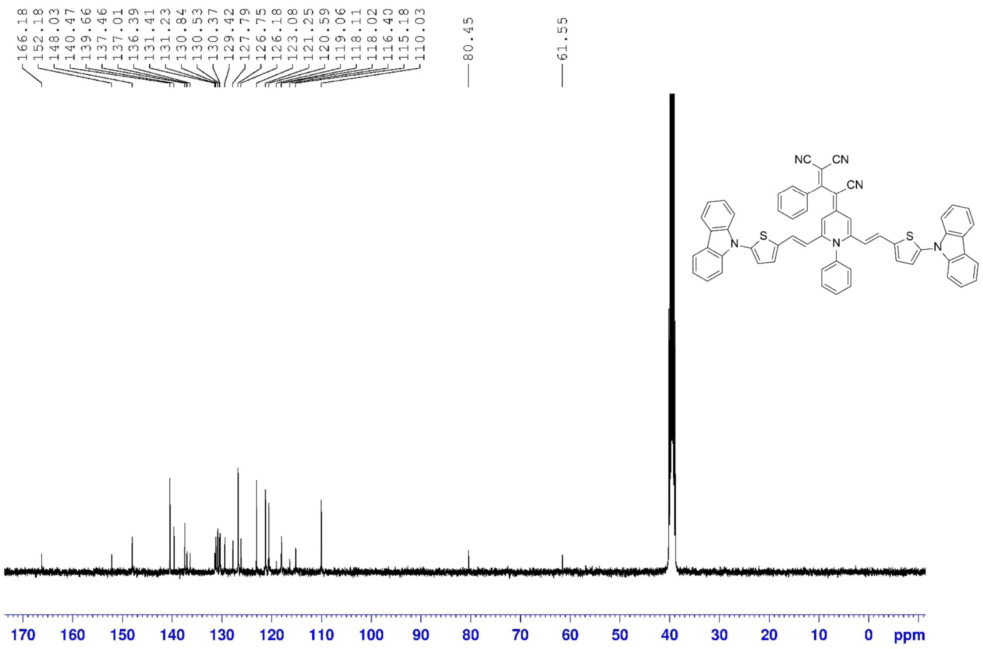

Figure S13. ^{13}C NMR spectrum of TCM-Ph.

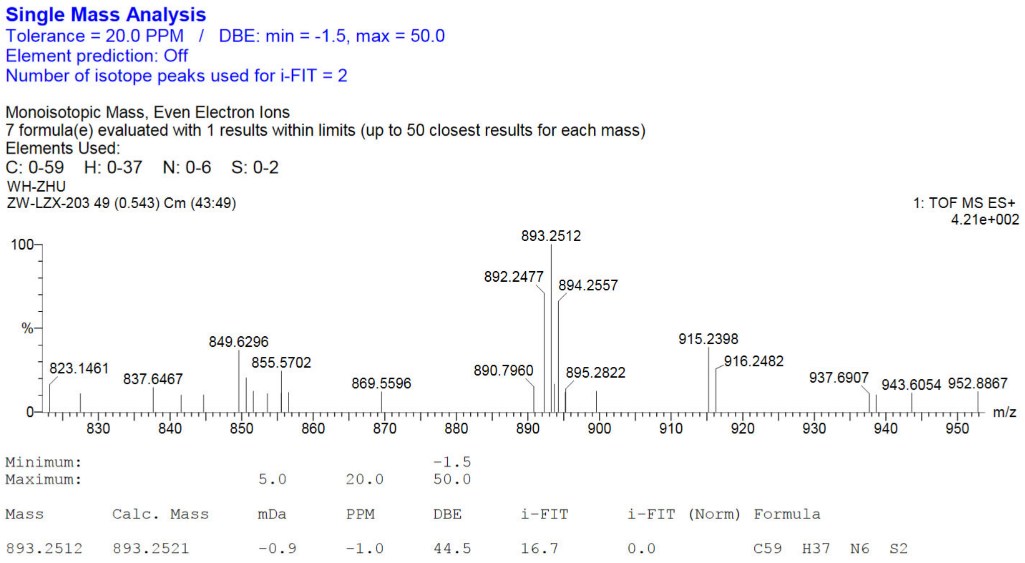

Figure S14. HRMS spectrum of of TCM-Ph.

References

36. Zhang, J.; Wang, Q.; Guo, Z.; Zhang, S.; Yan, C.; Tian, H.; Zhu, W. H. High-fidelity trapping of spatial–temporal mitochondria with rationaldesign of aggregation-induced emission probes. *Adv. Funct. Mater.* **2019**, 29, 1808153.
